# Treating children with disinhibited social engagement disorder symptoms: Filial therapy

**DOI:** 10.1192/j.eurpsy.2021.1700

**Published:** 2021-08-13

**Authors:** S. Hatam, S. Moss, C. Cubillo, D. Berry

**Affiliations:** 1 Health And Human Science, Charles Darwin University, Darwin, Australia; 2 Amsant, Aborginal Medical Services Alliance Northern Territory, Darwin, Australia; 3 Play Therapy, Play Therapy Australia, Perth, Australia

**Keywords:** Filial Therapy, attachment disorders, Disinhibited Social Engagement Disorder, play therapy

## Abstract

**Introduction:**

Children affected by social neglect and other forms of abuse are at significant risk of developing mental health problems as well as social, academic, and behavioral functioning difficulties. Some studies have assessed the effectiveness of treatment for children with trauma-attachment disorder. Nevertheless, some questions remain to be answered regarding appropriate treatment.

**Objectives:**

Aim This research identified how filial therapy affects the extent to which foster parents are responsive, sensitive, and attentive to the needs of their children in their care. Subsequently, the study explored how this bond, influenced during filial therapy, affects the signs and symptoms of disinhibited social engagement disorder.

**Methods:**

Method This study used case study as the methodology to research the influence of filial therapy (CPRT) in foster children who show the symptoms of disinhibited social engagement disorder- aged three to six. Two sets of foster parents received a 10-session filial therapy model (CPRT) across 10 weeks. Pre and post measures of the parent-child relationship were analyzed.

**Results:**

Result The findings indicate that filial therapy greatly enhances the bond between foster parents and children with DSEDs. Moreover, these improvements in the bond diminished the symptoms of disinhibited social engagement disorder.

**Conclusions:**

Conclusion The impact of filial therapy as a responsive intervention reduced the symptoms of disinhibited social engagement disorder. The symptoms have declined very likely as a result of rebuilding, regenerating, and enhancing the relationship between foster children and foster parents.

**Disclosure:**

No significant relationships.

